# Update on canine filaggrin: a review 

**DOI:** 10.1080/01652176.2020.1758357

**Published:** 2020-05-04

**Authors:** Daniel Combarros, Marie-Christine Cadiergues, Michel Simon

**Affiliations:** a UDEAR, Université de Toulouse, INSERM UPS, Toulouse, France; b Université de Toulouse, ENVT, Toulouse, France

**Keywords:** Canine, dog, atopic dermatitis, filaggrin, skin barrier, epidermis, genetics

## Abstract

Human filaggrin (FLG) plays a key role in epidermal barrier function, and loss-of-function mutations of its gene are primarily responsible for the development of human atopic dermatitis (AD). FLG expression is also reduced in the epidermis of atopic patients, due to the transcriptional effect of Th2 type cytokines. Canine atopic dermatitis (CAD) is a prevalent skin disease that shares many clinical and pathogenic features with its human homologue. The aim of this review is discuss current knowledge on canine filaggrin (Flg) in both healthy and atopic dogs, as compared to the human protein. Although the molecular structures of the two proteins, as deduced from the sequences of their gene, are different, their sites of expression and their proteolytic processing in the normal epidermis are similar. Concerning the expression of Flg in CAD, conflicting results have been published at the mRNA level and little accurate information is available at the protein level. It derives from a large precursor, named profilaggrin (proFLG), formed by several FLG units and stored in keratohyalin granules of the *stratum granulosum.* Canine and human proFLG sequences display little amino acid similarity (33% as shown using the Basic Local Alignment Search Tool (BLAST)) except at the level of the S100 homologous part of the N-terminus (75%). Genetic studies in the dog are at an early stage and are limited by the variety of breeds and the small number of cases included. Many questions remain unanswered about the involvement of Flg in CAD pathogenesis.

## 1. Introduction

The skin is an anatomical and physiological barrier between the environment and the organism. Keratinocytes, which originate at the basal layer of the epidermis, progressively evolve while crossing the spinous and granular layers towards the *stratum corneum* (SC), where corneocytes, the end products of terminal differentiation, accumulate; for reviews see (Nishifuji and Yoon 2013; Le Lamer et al. 2015). The SC plays key roles in cutaneous barrier function, and its impairment can lead to an increase in epidermal water loss and favors the penetration of environmental agents (allergens, chemicals, microorganisms, etc.) into the skin (Nishifuji and Yoon 2013; Le Lamer et al. 2015).

**Figure 1.**
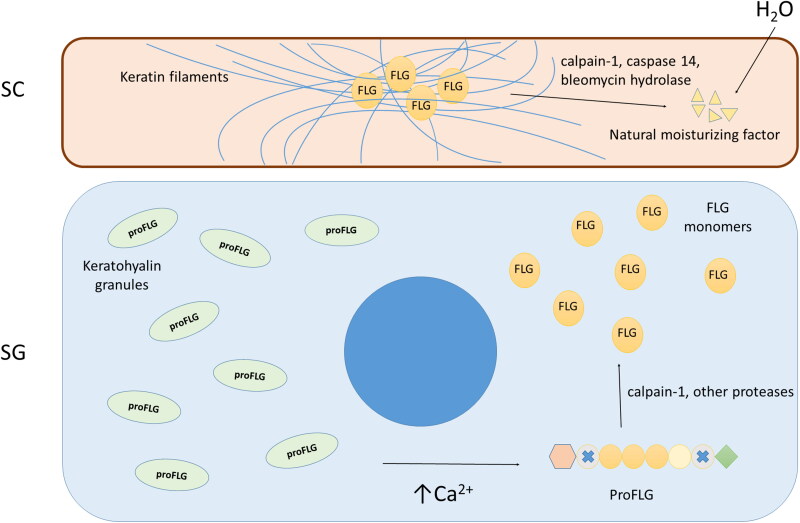
Schematic representation of filaggrin (FLG) metabolism. ProFLG, the FLG precursor, is stored in keratohyalin granules in the *stratum granulosum* keratinocytes. ProFLG is released in the cytoplasm in response to increased Ca^2+^ levels, then it is dephosphorylated and cleaved by not well-characterized proteases leading to the production of FLG monomers. The monomers aggregate keratin intermediate filaments and induce collapse and flattening of corneocytes. Finally, FLG is deiminated and degraded in the upper *stratum corneum* to natural moisturizing factor components. Abbreviations: SG, *stratum granulosum*; SC, *stratum corneum*; proFLG, profilaggrin; FLG, filaggrin. Adapted from Nishifuji and Yoon (2013).

Human filaggrin (FLG – *note to the reader: the abbreviation of the human gene is in upper case and the equivalent canine gene is in lower case*) plays a key role in epidermal differentiation and skin barrier function. It derives from a large precursor, named profilaggrin (proFLG), formed by several FLG units and stored in keratohyalin granules of the *stratum granulosum*; for reviews, see (Brown and McLean 2012; Henry et al. 2012). After release in the cytoplasm, in response to increased Ca^2+^ levels, proFLG is dephosphorylated and cleaved by proteases which leads to the production of FLG monomers. The monomers aggregate keratin intermediate filaments forming tight bundles, and induce the collapse and flattening of corneocytes, an essential feature of cornified layer formation (Brown and McLean 2012). Keratins and FLG are cross-linked to cornified envelopes, consolidating the structure. Ultimately, FLG is deaminated and totally degraded by several proteases in the upper SC to release its constitutive amino acids [part of the ‘natural moisturizing factor’ (NMF)] which contributes to epidermal hydration and barrier function (Figure 1) (Brown and McLean 2012; Henry et al. 2012).

In 2006, an association between loss-of-functions of the *FLG* gene and both atopic dermatitis (AD) and ichthyosis vulgaris was documented in Caucasians (Palmer et al. 2006; Smith et al. 2006), later confirmed in Asiatic populations (Thyssen and Kezic 2014), and finally identified in Afro-Americans (Margolis et al. 2018). Therefore, the prevalence of these diseases in the mutant population is significantly higher when compared to the non-mutant individuals (Palmer et al. 2006; Smith et al. 2006; Margolis et al. 2018). Today, *FLG* loss-of-function mutations are still the major genetic predisposing factor identified for the development of AD as individuals with these mutations are 3 to 5 times more likely to develop the disease (Thyssen and Kezic 2014). Since FLG is a key protein in epidermal barrier, this finding caused the scientific community to realise the importance of the skin barrier in AD (Brown and McLean 2012). These mutations result in a great reduction in FLG amount due to two processes: a very short proFLG molecule when the stop codon is close to the 5′ end of the coding sequence, and an unstable one when the stop codon is located in the 3′ part, resulting in the absence of the C-terminus that is needed for the processing of proFLG to FLG (Brown and McLean 2012).

Human and canine atopic dermatitis (CAD) are both prevalent in chronic inflammatory and pruritic skin diseases (Marsella and De Benedetto 2017). They share many clinical and pathogenic features, such as increasing prevalence of the disease over time, the fact that similar areas of the body are preferentially affected (including the extremities, face, neck and chest), a reduction of skin microbial diversity, an abnormal immune response to environmental antigens or an epidermal barrier impairment due to alterations in lipid composition (Marsella and De Benedetto 2017). Because of the similarities between the two diseases, the dog could constitute a valuable model for human AD. However, less knowledge exists in the veterinary literature concerning CAD and the canine model is not yet fully characterized.

It is important to understand that the environment plays a key role, not only in the development of the atopic disease but also directly in FLG expression (Thyssen and Kezic 2014). Increase in the external relative humidity and exposure to the sun or irritants can reduce epidermal FLG levels and lead to an acquired filaggrin deficiency (Thyssen and Kezic 2014; Cau et al. 2017). An inflammatory Th2 impregnation, as observed in AD skin, also reduces FLG synthesis (Table 1) (Pellerin et al. 2013). The FLG deficiency, be it genetically determined or acquired, causes an altered epidermal structure and an impaired barrier function that favors penetration of environmental allergens into the skin, including house dust mite, pollen, bacteria and fungus components, thus enhancing sensitization of the individual (Brown and McLean 2012). Furthermore, the consequent alteration of the physico-chemical properties of the epidermis (increased pH, altered lipid secretion, modification of intracellular and extracellular architecture of keratinocytes, reduction of antimicrobial peptides, etc.) favors the proliferation of microorganisms like bacteria and fungi culminating in the recurrent skin infections that are observed in both human and canine AD patients (Thyssen and Kezic 2014; Marsella and De Benedetto 2017).

**Table 1.** Environmental and inflammatory factors known to alter the amount of human epidermal profilaggrin, filaggrin and/or filaggrin-derived natural moisturizing factor components (adapted from Thyssen and Kezic (2014)).

Environmental factors*
Humidity level
UV-B radiation
Mechanical damage (scratching, tape stripping)
Ageing
Skin irritants (sodium lauryl sulfate, etc.)
Psychological stress
Inflammatory factors*
IL-4, IL-13, IL-17A, IL-22, IL-25, IL-31, TNF-α

* The change in the amount of profilaggrin, filaggrin and/or natural moisturizing factor can be the consequence of either regulation of *FLG* gene expression or alteration in the expression/activity of enzymes involved in profilaggrin proteolytic maturation and filaggrin degradation (peptidylarginine deiminases, calpain-1, caspase 14, bleomycin hydrolase, etc.).

In the next parts of this review we present the current knowledge on canine filaggrin (Flg). Available data about the association between Flg and CAD will be discussed.

## 2. Profilaggrin structure in humans and dogs

**Figure 2.**
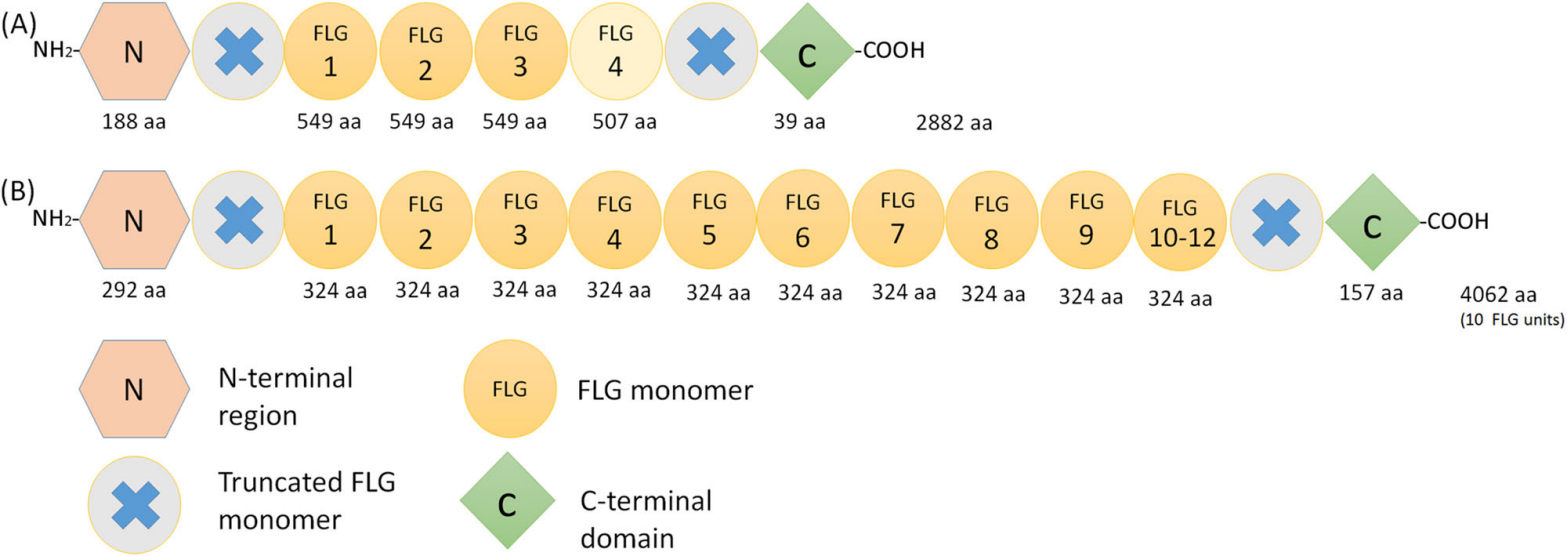
Schematic representation of the proFilaggrin protein. (A) in dog and (B) in man. Note the differences in the number of filaggrin (FLG) repeats and in the number of the amino acids that form the FLG monomers and the C and N‐terminal domains (Brown and McLean 2012; Kanda et al. 2013; Pin et al. 2019). Abbreviations: aa, amino acid; FLG, filaggrin.

In dogs, the *Flg* gene is located on chromosome 17, within the so-called epidermal differentiation complex (Mischke et al. 1996). The encoded protein forms part of the S100 fused-type protein family together with filaggrin-2 (Flg2), trichohyalin, hornerin and others (Mischke et al. 1996; Henry et al. 2012). As in humans (Brown and McLean 2012; Henry et al. 2012), the proFlg molecule comprises a N-terminal region, with 2 calcium-binding sites, a C-terminal region and a central region, which contains repeated Flg monomers (Figure 2) (Kanda et al. 2013). However, there are major structural differences between the precursors in the two species. The number of *FLG* repeats varies from 10 to 12 in humans (Brown and McLean 2012) and is only 4 in dogs (Kanda et al. 2013; Pin et al. 2019). The number of amino acids composing the monomers and the C- and N-terminal domains are also substantially different between the species. In dogs, two different Flg monomers are present, one composed of 549 amino acids, which is repeated three times, and a smaller, 507 amino acid long monomer present only once (Kanda et al. 2013). In humans, all monomers are essentially identical in length (324-325 amino acids) (Figure 2) (Brown and McLean 2012).

Canine and human proFLG sequences display little amino acid similarity (33% as shown using the Basic Local Alignment Search Tool (BLAST); the authors’ unpublished data) except at the level of the S100 homologous part of the N-terminus (75%). Additionally, whereas the sequences of human FLG repeats display 40% amino acid sequence variability, there are only rare changes in the sequences of canine Flg monomers (Kanda et al. 2013; Pin et al. 2019).

## 3. Expression pattern and localization of Flg in normal dogs

In healthy dogs, proFlg is located in keratohyalin granules of the *stratum granulosum* cells, and Flg in the matrix of the lower SC (Kanda et al. 2013; Pin et al. 2019). No labelling is observed in the upper SC, probably because of the total degradation of Flg and the formation of NMF. This distribution is similar to what has been described in human skin (Pellerin et al. 2013). This localization pattern was first evidenced using immunohistochemistry (IHC) (Kanda et al. 2013; Pin et al. 2019) and subsequently confirmed by post-embedding immunoelectron microscopy (Pin et al. 2019). One study, using IHC (Theerawatanasirikul et al. 2012), reported that patterns of Flg expression were similar in different breeds (poodles, golden retrievers, shiz tzus, pugs and labradors) and body locations (16 different regions). However, this result is questionable in view of the pan-epidermal staining pattern obtained, suggesting that the rabbit polyclonal anti-FLG antibody used was not specific for canine Flg.

In western-blot assays, two bands, of 59 and 54 kDa, corresponding to the two different predicted dog Flg monomers, were found (Kanda et al. 2013; Pin et al. 2019). Additionally, a band of 250 kDa that probably corresponded to canine proFlg, and some intermediate bands suggestive of proFlg processing were also observed (Pin et al. 2019).

## 4. Flg expression and CAD

Limited accurate information exists concerning how Flg expression is modified in CAD. IHC staining intensity in the epidermis from 11 atopic and 4 healthy Beagle dogs was lower, both at baseline and after allergen challenge exposure (Marsella et al. 2009). However, this result remains questionable; indeed all the epidermal layers were immunostained, showing that the anti-Flg antibody used was non-specific and cross-reacted with additional keratinocyte proteins. In another study, skin biopsies from 18 atopic and 16 control dogs from 12 different breeds were analyzed with an antibody directed against the C-terminal region of the proFlg molecule (Chervet et al. 2010). Lack of labelling was observed in the skin of four of the atopic dogs whereas Flg was similarly immunodetected in the epidermis of the other atopic and all the control dogs. This finding suggests the existence of a possible loss-of-function mutation in *Flg* gene in some dogs, as seen in humans.

Conflicting results have been obtained concerning *Flg* mRNA expression in healthy and atopic dogs. In one study, *Flg* expression was found to be decreased in atopic nonlesional skin as compared to control skin. However, the downregulation was statistically significant only when the analysis was limited to West Highland white terrier dogs and not when all animals included in the study were considered (Roque et al. 2011). Conversely, other authors found a statistically significant upregulation in lesional atopic skin and a trend to upregulation in nonlesional atopic skin, as compared to healthy control skin (Theerawatanasirikul et al. 2012). These studies have two major limitations: the wide variety of canine breeds included and the fact that the dogs were client owned (likely living in very different environmental conditions and having lesions for variable durations). In a study that challenged sensitized non-atopic beagles with allergen using a *Dermatophagoides farinae* (*Df*) extract, no significant changes in *Flg* mRNA expression were observed in skin biopsies (Schamber et al. 2014).

Alteration in Flg metabolism has also been suggested. Several proteases are involved in the processing of proFLG to FLG monomers, including calpain-1, and in the degradation of FLG monomers leading to the NMF (caspase-14, calpain-1, bleomycin hydrolase, etc.) (Brown and McLean 2012; Henry et al. 2012; Le Lamer et al. 2015). In a study including 8 healthy and 8 atopic colony dogs, biopsies from non-lesional atopic skin showed a marked decrease in caspase-14 staining (Marsella et al. 2016). However, in a second study from the same group, including 4 healthy and 4 atopic dogs, higher expression of calpain-1, caspase-14 and matriptase was observed in nonlesional atopic skin. No differences were observed after allergen challenge between lesional atopic and healthy skin at days 3, 10, 14 or 28 (Fanton et al. 2017). It is unknown whether this down or up-regulation of enzymes involved in Flg metabolism has any consequences for Flg degradation and NMF production, since Flg expression has not been studied in parallel. Further studies are needed to clarify the discrepancies and to better characterize the expression and activity of these enzymes in relation with CAD.

When an altered Flg expression is observed, whether this change is primary or secondary to inflammation remains a matter of debate (Fanton et al. 2017). As in humans, treatment of canine reconstructed epidermis with Th2 type cytokines induces a marked decrease in Flg immunodetection (Pin et al. 2019).

## 5. Filaggrin 2

FLG2, another S100 fused-type protein, shares several properties with FLG in terms of structural organization, cellular localization and amino acid composition (Wu et al. 2009). Functionally, FLG2 seems to evolve to form the cornified envelope and NMF in equal proportions, whereas most FLG is degraded to form NMF (Hsu et al. 2011; Albérola et al. 2019). The first half of FLG2 is cross-linked to the envelope whereas the second half is degraded in the upper SC (Albérola et al. 2019). To the best of the authors’ knowledge, FLG2 has not been characterized in dogs as it has been in humans (Wu et al. 2009).

Some studies in veterinary medicine have focused on Flg2 expression in the context of CAD. One study concerned a colony of atopic beagles sensitized and challenged with *Df* extracts (Marsella 2013). Using IHC, it showed no correlation between Flg2 expression and the severity of clinical signs at different time points (d0, d3 and d10) (Marsella 2013). Another study with a similar protocol showed that the administration of a probiotic containing *Lactobacillus rhamnosus* did not alter Flg2 expression before or after allergen challenge with *Df* extracts when compared to the non-supplemented atopic controls (Marsella et al. 2013). Flg2 mRNA expression increased in the skin of atopic beagles relative to healthy controls after challenge with *Df extracts* at d3 and d10 (Santoro et al. 2013). This increased amount of Flg2 mRNA was not accompanied by an increase in Flg2 protein (assayed using IHC), thus suggesting an increase in protein degradation or post-transcriptional alteration (Santoro et al. 2013). Finally, in an experimental model of CAD, *Flg2* mRNA expression, but not *Flg*, was downregulated following allergen exposure (Schamber et al. 2014).

## 6. Associations between *Flg* mutations and CAD

Several thousands of people have been studied to highlight the FLG-AD association (Palmer et al. 2006). This large number of samples is necessary to detect any genetic association with a polygenic disease, especially when the association is weak. However, such studies are very difficult to perform in veterinary medicine. On the other hand, identification of disease related SNPs in dogs should be easier because of the presence of highly inbred genetic isolates. It is well known that *FLG* loss-of-function mutations show population specificities in humans, 80% of the *FLG*-null alleles in the white European population consisting of only two mutations, whereas, in the Singaporean Chinese population, there are eight different *FLG*-null mutations that account for 80%. In Afro-Americans, population-specific mutations have been revealed (Margolis et al. 2018). Furthermore, two of the most prevalent *FLG* mutations in the north of Europe, which are present in around 7% of the normal population, are present in less than 1% of the Italian population (Brown and McLean 2012). This highlights the importance of performing geographically restricted studies.

The first study investigating the association of *Flg* mutations with CAD included dogs from 8 different breeds and three geographically distant regions (UK, USA and Japan) (Wood et al. 2010). Despite the large number of dogs included (242 atopic and 659 control dogs), their very different genetic backgrounds made the interpretation of results difficult. Nevertheless, the authors found a significant association between a single nucleotide polymorphism (SNP) (rs22588227) in the *Flg* gene and CAD in UK Labradors (23 atopic and 75 controls) with an odds ratio of 5.6 (Wood et al. 2010). This association was not replicated when USA Labradors were included (33 atopic and 43 controls), which emphasizes the role of the geographic location in genetics even when samples come from a single breed (Wood et al. 2010). This SNP has not been studied further to determine its location in the *Flg* gene or whether it is at the origin of either a modification of the amino acid sequence or a stop codon. The small numbers of UK Labradors increased the risk of false positive association but the high odds ratio makes some relationship probable (Wood et al. 2010).

Three studies focusing on West Highland white terrier dogs, this time in more geographically restricted areas, failed to document any link of CAD with the *Flg* gene (Barros Roque et al. 2009; Salzmann et al. 2011; Roque et al. 2012). The first included 49 atopic and 30 control dogs and was performed in Australia (Barros Roque et al. 2009); the second recruited animals across the USA and included 90 dogs (22 affected, 40 healthy and 28 of undetermined status) belonging to three family groups (Salzmann et al. 2011); and the third included 35 atopic subjects and 25 controls, again from Australia (Roque et al. 2012). The USA study may include an inclusion bias because the animals were selected only by reviewing a medical health questionnaire (Salzmann et al. 2011). Dogs were sometimes very young (one year of age), so signs of clinical AD may not yet evidence in these animals (Salzmann et al. 2011). In the third study, an association of CAD with a 1.3-Mb region on chromosome 17 (in which the gene coding for *Flg* is located) was observed (Roque et al. 2012). However, the peak association signal was located more than 10 Mb from the canine *Flg* gene, thus excluding any association with a *Flg* mutation (Roque et al. 2012). Although a weak association may have been missed because of the small number of dogs included in these three studies, a large causative role for the canine *Flg* gene in AD in this breed is unlikely (Barros Roque et al. 2009; Salzmann et al. 2011; Roque et al. 2012).

Although other SNPs have been found to be associated with AD in different breeds in Thailand, the small number of animals included in the study (8 poodles, 2 shih tzus and 2 pugs in the atopic group and 13 poodles, 15 shih tzus and 1 pug in the control group) limits the interpretation of the results (Suriyaphol et al. 2011).

Interestingly, a recent case report described a new form of ichthyosis in a German Shepherd dog, which was attributed to a *de novo* missense mutation in the gene *ASPRV*1 encoding aspartic peptidase retroviral-like 1 (SASPase) (Bauer et al. 2017). This enzyme is suspected to be involved in the processing of proFlg to Flg. This case report suggests that, in the dog, a loss of function mutation of the *Flg* or processing related enzymes could manifest itself by an ‘ichthyosis’ phenotype as is seen in humans with loss of function mutations of the *FLG* gene (ichthyosis vulgaris) (Bauer et al. 2017). Additionally, deletion of the gene of another protease involved in FLG degradation, namely caspase-14, has been identified as the cause of a rare human autosomal recessive congenital ichthyosis (Kirchmeier et al. 2017). Conversely, we could expect *ASPRV1* gene polymorphisms to be a predisposing genetic factor to AD development in humans and dogs; this is something that could be looked at in the future.

Except for what is known in UK Labrador Retrievers and West Highland white terrier dogs (Barros Roque et al. 2009; Wood et al. 2010; Salzmann et al. 2011; Roque et al. 2012), the question of *Flg* mutations and their association with CAD remains unanswered. The wide diversity of dog breeds, the difficulty of collecting samples from large numbers of diseased and control animals in a specific geographic area, the financial cost of sample collection and processing, may result in this concern remaining unsolved for a long time.

## 7. Conclusion

CAD is a very complex multifactorial polygenic disease with high inter-breed differences and is thus a complex subject. As mentioned above, the Flg gene and the pattern of expression of the protein in healthy dogs are well characterized. However, limited information is available concerning the role, if any, of Flg in CAD. Although the number of publications is relatively large, major technical limitations make results difficult to interpret.

In studies about CAD, special attention should be paid to inclusion criteria. Small numbers of animals from different breeds and various environments are not suitable for studying such a complex disease. Based on the difficulty of performing large-scale studies in veterinary medicine, the analysis of a colony of dogs with a spontaneous form of AD and a colony of control dogs with similar genetic background and similar housing conditions is the only option to obtain interpretable results and to progress in the understanding of this disease.

Unfortunately, commercial antibodies specifically directed against dog epidermal proteins are largely lacking. Although some proteins are very well conserved between species, major sequence differences may exist in others, as in canine and human FLG. Antibodies raised against human, mouse or other species proteins need to be thoroughly validated before being used in dogs if accurate results are to be obtained.

## Abbreviations

CAD: canine atopic dermatitis
*Df*: *Dermatophagoides farinae*
FLG: filaggrin (human protein);
*FLG*: filaggrin (human gene)
Flg: filaggrin (dog protein)
*Flg*: filaggrin (dog gene)
IHC: immunohistochemistry
SNP: single nucleotide polymorphism
